# Effects of Oral L-Carnitine Supplementation on Lipid Profile, Anemia, and Quality of Life in Chronic Renal Disease Patients under Hemodialysis: A Randomized, Double-Blinded, Placebo-Controlled Trial

**DOI:** 10.1155/2012/510483

**Published:** 2012-06-05

**Authors:** Afsoon Emami Naini, Mahnaz Moradi, Mojgan Mortazavi, Asghar Amini Harandi, Mehdi Hadizadeh, Farhad Shirani, Hamed Basir Ghafoori, Pardis Emami Naini

**Affiliations:** ^1^Isfahan Kidney Diseases Research Center, Isfahan University of Medical Sciences, Isfahan 8168798847, Iran; ^2^Biochemistry Department, School of Medicine, Jahrom University of Medical Sciences, Jahrom 7414846199, Iran; ^3^Department of Emergency Medicine, Sina Hospital, Tehran University of Medical Sciences, Tehran 1473115447, Iran

## Abstract

In patients on maintenance hemodialysis several factors reduce the body stored carnitine which could lead to dyslipidemia, anemia, and general health in these patients. We evaluated the effect of oral L-carnitine supplementation on lipid profiles, anemia, and quality of life (QOL) in hemodialysis patients. In a randomized, double-blinded, placebo-controlled trial, end-stage renal disease (ESRD) patients on hemodialysis received either L-carnitine 1 g/d (*n* = 24) or placebo (27 patients) for 16 weeks. At the end of the study, there was a significant decrease in triglyceride (−31.1 ± 38.7 mg/dL, *P* = 0.001) and a significant increase in HDL (3.7 ± 2.8 mg/dL, *P* < 0.001) levels in the carnitine group. Decrease in total cholesterol (−6.6 ± 16.0 mg/dL, *P* = 0.075) and increase in hemoglobin (0.7 ± 1.7 g/dL, *P* = 0.081) concentrations in the carnitine group were not significant. There was no statistically significant changes in LDL in any group (*P* > 0.05). Erythropoietin dose was significantly decreased in both the carnitine (−4750 ± 5772 mg, *P* = 0.001) and the placebo group (−2000 ± 4296 mg, *P* < 0.05). No improvement was observed in QOL scores of two groups. In ESRD patients under maintenance hemodialysis, oral L-carnitine supplementation may reduce triglyceride and cholesterol and increase HDL and hemoglobin and subsequently reduce needed erythropoietin dose without effect on QOL.

## 1. Introduction

The prevalence and incidence of chronic kidney diseases (CKDs) is increasing all over the world and in Iran as well [1]. In spite of medical advancements and increased survival rate of the patients, atherosclerosis and the subsequent cardiovascular diseases are still the most important causes of mortality in CKD patients and end-stage renal disease (ESRD) [2, 3].

Dyslipidemia is commonly observed in ESRD patients, which is one of the major underlying causes of development and progression of atherosclerosis in this group of patients. Almost 40% of patients on continuous hemodialysis have some types of dyslipidemia, and most of them are affected by hyperlipidemia type IV and decreased high-density lipoprotein (HDL) plasma level [3]. Development and progression of dyslipidemia in these patients is influenced by various factors such as carnitine deficiency which could lead to abnormalities in lipid metabolism [4, 5]. Carnitine or trimethyl-aminobutyric acid is a natural and essential vitamin-like substance for human body. It is involved in many metabolic processes including regulation of ketogenesis, conformation and control of mitochondrial energy, and transportation of long-chain free fatty acids from cytoplasm into mitochondria for beta-oxidation. Therefore, presence of sufficient amounts of carnitine in cells is essential for normal oxidation of fatty acids. The substance is of great importance in tissues such as cardiac or skeletal muscles, which rely on fatty acid metabolism for energy production [6]. Moreover, several metabolic disorders such as oxidative stress and transformation of phospholipids that are caused following carnitine deficiency can affect the development and progression of anemia as another commonly observed complication in patients with ESRD [7].

Since the kidney is one of the main sites of carnitine synthesis, endogenous carnitine production is significantly reduced after the kidney parenchymal damage [5]. Moreover, since carnitine is synthesized from lysine and methionine in human body, the body requires exogenous carnitine to achieve a daily balance of carnitine. Meat and milk products are rich sources of exogenous carnitine and its precursors [6]. Nevertheless, poor nutritional state is common in patients with hemodialysis [8]. Thus, patients under hemodialysis receive limited amounts of food containing carnitine and its precursors. Also, the cofactors and carnitine precursors such as vitamin B6, niacin, vitamin C, lysine, and methionine may be removed during hemodialysis process [5]. The small molecule of carnitine is very polar and water-soluble and can freely pass the dialysis membrane, such that in each hemodialysis session, the serum level of carnitine is decrease by 75% [5].

Several studies have been conducted on the effects of carnitine supplement administration on dyslipidemia, skeletal muscle function, cardiac function, and anemia in patients under continuous hemodialysis. The effect of carnitine supplementation on dyslipidemia has been remained unknown [9]. In addition, there is also a lack of data on the effects of carnitine supplementation on quality of life (QOL) in ESRD patients.

Considering the possible role of carnitine deficiency in development of dyslipidemia in ESRD and contrary findings of previous studies in this respect, this study was carried out to determine the effect of oral L-carnitine supplement on lipid profile, anemia, and life quality of dyslipidemic ESRD patients under continuous hemodialysis.

## 2. Methods and Materials

### 2.1. Patients and Setting

This randomized, placebo-controlled, double-blinded, clinical trial was carried out on dyslipidemic ESRD patients under continuous hemodialysis in Noor and Al-Zahra University Hospitals in Isfahan, Iran. The inclusion criteria were the age range of 18–75 years, history of at least 12 weeks of hemodialysis, three times a week and each session almost 4 hours, and serum TG or total cholesterol concentration >200 mg/dL or serum HDL concentration <40 mg/dL at the beginning of the study. The patients were not included into the study if they were on carnitine supplement or any drug that interacts with carnitine, that is, anticoagulant medication or those lowering the seizure threshold (e.g., tricydic antidepressants) in the last month. Patients on other drugs influencing lipid metabolism, such as *β*-blockers, glucocorticoids, or lipid-lowering agents during the previous eight weeks were not enrolled. Furthermore, patients with liver dysfunction, hypothyroidism, chronic infectious diseases such as hepatitis, active source of infection or inflammatory diseases, or history of seizure and known cases of any brain mass according to medical history were not included. Sampling was performed with convenient nonrandomized method from among the patients referred to the dialysis ward of the hospitals. Considering the study power of 80%, type one error of 5%, and at least 5 mg/dL increase in HDL concentration, according to the previous studies [7], and also with respect to 10% of dropouts, the sample size for each group was determined to be 25. The study was approved by the Ethical Committee of Isfahan University of Medical Sciences, registered in http://www.clinicaltrial.org/ *(NCT01278693)*, and all patients signed a fully informed written consent before including to the study.

### 2.2. Intervention

Eligible patients were assigned to either carnitine or placebo groups based on the random number table, generated by random allocation software [10]. The patients in the carnitine group received 1 g of carnitine oral supplement each day (two 250 mg tablets twice a day, for 16 weeks), and the patients in the placebo group received the placebo mad by the same company in the same manner and duration. L-carnitine and placebo were coded and the physician, researchers, and patients were blinded to the code of carnitine and the placebo. The patients were followed up for drug side effects and the compliance for drug consumption every two weeks in the treatment centers. In case of occurrence of severe complications associated with carnitine including severe gastrointestinal upset, muscle weakness, and seizure or discontinuation of the drug for more than one week, the subject was excluded from the study.

### 2.3. Clinical and Laboratory Evaluations

Demographic characteristics, including age, sex, body mass index (BMI), the underlying cause of ESRD, and duration of dialysis were recorded at the beginning of the study. Moreover, total cholesterol, HDL, low-density lipoprotein (LDL), TG, and hemoglobin (Hb) serum concentrations, as well as the received erythropoietin dose was recorded at the onset of the study and 8 and 16 weeks after the beginning of the intervention. To assess the QOL of patients, we used the Short-Form Health Survey (SF-36) [11] questionnaire at the beginning, and 8 and 16 weeks after the beginning of the study.

### 2.4. Statistical Analysis

To compare baseline and demographical characteristics of the participants between the two groups, we used chi-square and independent sample *t*-test. For evaluation of variable changes in the two groups, we used repeated measures and paired *t*-test. Multivariate analysis was done for controlling covariate factors. The data was analyzed using SPSS software, version 16.0 (SPSS Inc., Chicago, IL, USA). The *P* value less than 0.05 considered statistically significant.

## 3. Results

Twenty-seven patients were entered into each group. In the carnitine group, three patients were excluded; one underwent kidney transplantation, one died because of myocardial infarction, and one was not willing to continue the study. Thus, we analyzed the information of 24 patients in the carnitine group and 27 patients in the placebo group. As it is reported in Table 1, two groups were not significantly different with respect to demographical or baseline characteristics, except for BMI which was higher in the carnitine group (*P* = 0.383). We omitted two extra value cases of obesity in carnitine group, and analysis was repeated again but there were not significant changes in final result comparing to the previous analysis.

Of total, 5 patient in carnitine group and 6 patients in placebo group had high triglyceride (TG), high cholesterol, and low HDL levels. There was no difference between baseline TG and Hb levels in 2 groups (TG: *P* = 0.204; Hb: *P* = 1.84). The changes in lipid profile, Hb, and received dose of erythropoietin are shown in Table 2. Also, comparison of the amount of changes in these variables between the two groups is presented in Table 3. The concentration of TG was significantly decreased in the carnitine group (−31.1 ± 38.7 mg/dL, *P* = 0.001), while it was increased in the placebo group (9.6 ± 32.2 mg/dL, *P* = 0.029). Decrease in cholesterol level in the carnitine group was not statistically significant (−6.6 ± 16.0 mg/dL, *P* = 0.075 for repeated measure test and = 0.055 for paired *t*-test), and there was no significant change in the placebo group as well (−3.6 ± 24.9 mg/dL, *P* = 0.517). The HDL concentration in the carnitine group increased significantly by 3.7 ± 2.8 mg/dL (*P* < 0.001), but it did not change significantly in the placebo group (*P* = 0.185). The LDL concentration, however, did not change in any group (*P* = 0.237 in the carnitine group; *P* = 227 in the placebo group). According to repeated measure analysis, the changes in Hb level was not statistically significant in the carnitine group (*P* = 0.081), but with paired *t*-test, there was a significant increase in Hb level after the study (*P* = 0.037). No significant change was observed in Hb level in the placebo group (*P* = 0.145). The required dose of erythropoietin significantly decreased in both groups, by a greater amount of decrease in the carnitine group (−4750 ± 5772; *P* = 0.001 versus −2000 ± 4296 U/Wk; *P* = 0.033).

The changes of overall scores of QOL and its physical and mental subscales in the two groups are presented in Table 4. Also, comparison of the changes between the two groups is demonstrated in Table 5. The overall QOL score changed significantly in both groups (*P* = 0.148 in carnitine group; *P* = 0.087 in placebo group). Score of the physical health domain decreased in the carnitine group (*P* = 0.033), and score of the mental health domain decreased in the placebo group (<0.001).

Considering differences between the two groups in BMI and baseline lipid profiles, we conducted multivariate analysis to determine the effect of carnitine on each of the lipid profile components controlling by age, sex, BMI, duration of hemodialysis, and baseline level of the related variable. According to the results, baseline level of TG (*t* = 6.2, *P* < 0.001), receiving carnitine (*t* = 2.2, *P* = 0.040), and female gender (*t* = 4.8, *P* < 0.001) significantly predicted decrease in TG level after study. For decrease in cholesterol level after study, baseline cholesterol level (*t* = 2.4, *P* = 0.025) and duration of hemodialysis (*t* = 3.7, *P* = 0.002) were predictors, but receiving carnitine did not significantly predict changes in cholesterol level (*t* = 1.7, *P* = 0.095). Baseline HDL level (*t* = −3.4, *P* = 0.003), BMI (*t* = −4.1, *P* = 0.001), male gender (*t* = −4.1, *P* = 0.001), and receiving carnitine (*t* = 3.3, *P* = 0.004) were predictors of increase in the HDL level after study. Only duration of hemodialysis was a predictor for lower LDL level after study (*t* = 2.8, *P* = 0.012) and receiving carnitine was not associated to changes in LDL level (*t* = 0.298, *P* = 0.769).

## 4. Discussion

Our results indicated that L-carnitine decreased the concentrations of TG and significantly increased HDL concentration. The decrease in cholesterol level was not statistically significant which might be due to the small sample size. L-carnitine did not have a significant effect on LDL concentration.

It is well known that carnitine involves in many metabolic processes. There is a negative correlation between serum free carnitine and total TG levels in iron-deficient patients [12]. It has been shown that carnitine could significantly reduce TG in plasma and tissues of sedentary rats and intake of hydrogenated fat diet in rats significantly increased the plasma and tissue lipid profile and monounsaturated fatty acids-rich diet or carnitine supplementation and/or exercise may ameliorate the deleterious effects of hydrogenated fat [13].

Carnitine supplementation further augmented the oxidative capacity of both liver and muscle significantly by enhancing the activity of carnitine palmitoyl transferase and the respiratory chain enzymes. These effects can be attributed to the enhanced unsaturated fatty acids in phospholipids of mitochondria and may be due to increased fluidity of the membrane in these rats [14].

Previous studies reported different effects of carnitine supplementation on lipid profile. Similarly to our results, the results of a meta-analysis showed that carnitine supplementation in patients on hemodialysis leads to a decrease in TG and cholesterol levels and also an increase in HDL level, without any significant change on the LDL level [15]. Nevertheless, another systematic review on carnitine supplementation did not report significant effect of carnitine on TG, cholesterol, or its derivatives [9]. The heterogeneity of the results of clinical trials can be explained by the difference of the studies with respect to baseline lipid profile status, the administered dose of carnitine supplement, the route of administration (oral or venous), and the specific characteristics of population under evaluation [9]. In some studies, the carnitine supplement was effective for modification of lipid profile of the patients with baseline TG level more than 200 mg/dL or HDL level less than 35 mg/dL [7]. Accordingly, we carried out our intervention on such patients, and our results indicated positive effect of carnitine on lipid profile. However, multivariate analysis in our study showed that carnitine decreased the TG level and increased the HDL level regardless of the baseline levels of each variable. In a recent randomized placebo-controlled trial, the efficacy of carnitine therapy was studied. After 3 months, serum level of lipoprotein(a) reduced significantly but serum level of TG and other lipoproteins did not significantly alter. However, they used carnitine as add-on therapy with atorvastatin or lovastatin to assess the efficacy of supplement therapy [16].

The effect of carnitine on anemia of patients under continuous hemodialysis was evaluated in previous studies, and the results indicated that carnitine supplementation enhances the response to administered dose of erythropoietin in these patients, which ends in increased hematocrit, reduced dose of required erythropoietin, and decreased index of erythropoietin resistance [7]. In our study, L-carnitine supplementation increased hemoglobin concentration. Although the required dose of erythropoietin significantly decreased in both groups, the amount of decrease was greater in the carnitine group.

The effect of L-carnitine on QOL of ESRD patients is an important topic which received less attention in previous studies. In a randomized, placebo-controlled, cross-over design trial, Semeniuk and colleagues used intravenous L-carnitine (20 mg/kg) comparing to placebo after each dialysis session for 12 weeks. But there was no significant effect on QOL. It is of note that length of study was less than our trial. Also the route, interval, and dose of prescription were different from our study. In addition they used specific inclusion criteria containing intradialytic hypotension, muscle cramping, lack of energy, muscle weakness or myopathy, cardiomyopathy, or lack of responsiveness to erythropoietin. The considered criteria are important direct consequences and comorbidities in patients with ESRD on chronic hemodialysis that can influence on QOL [17]. In another study, the SF-36 questionnaire was used, and administration of L-carnitine for eight weeks led to 18.2 points increase in the total QOL score [18]. In a controlled trial, after six weeks of administration, L-carnitine supplementation led to positive effects on QOL, but after 24 months of supplementation, the effect was not detectable [19]. We observed that L-carnitine supplementation did not have a considerable effect on the overall score of QOL of the patients. Considering the reported effects of L-carnitine on muscular weakness, cardiac function, and anemia, positive effects on the patients' physical health were expected, but conversely we observed about four-point decrease in the score of this domain in the carnitine group. The score of mental health domain of QOL significantly decreased in the placebo group, but the change in the carnitine group was not significant.

It should be considered that L-carnitine has some anti-inflammatory effect that can influence on our results. In a study by Savica et al., maintenance hemodialysis patients were assigned to receive intravenous injections of L-carnitine 20 mg/kg comparing to placebo thrice weekly at the end of each hemodialysis treatment for 6 months. The carnitine-treated group showed a statistically significant decrease in serum C-reactive protein and increase in serum albumin and transferrin, blood Hb, and body mass index. They reported that in hemodialysis patients, L-carnitine therapy may suppress inflammation, particularly among those patients with C-reactive protein ≥3 mg/dL, and may improve protein-energy nutritional status [20]. Although we did not monitored inflammatory biomarkers in this group of patients, we excluded patients with evidence of active infection diseases or history of inflammatory diseases. However, we connot discuss the possible role of inflammation and anti-inflammatory effects of L-carnitine on lipid profile, anemia and QOL in hemodialysis patients.

Our study has some limitations. The randomization was not effective to match patients in both groups in the case of BMI and lipid profile. Furthermore, because of the small sample size, a precise multivariate analysis to evaluate the predictive factors of response to treatment was not possible. Some possible contributory factors such as folate and iron were not measured.

## 5. Conclusion

In dyslipidemic ESRD patients under continuous hemodialysis, oral L-carnitine supplementation can decrease TG and increase HDL levels, without significant effects on cholesterol or LDL levels. In spite of the observed positive effects of oral L-carnitine on lipid profile and anemia, it did not have a positive effect on the patients' QOL in our study. Complementary studies with larger sample size, longer follow up, and evaluation of other important treatment outcomes such as cardiopulmonary function and the kidney disease specific QOL are necessary.

## Figures and Tables

**Table 1:** Demographic characteristics in two groups.

		Carnitine	Placebo	*P*
		*n* = 24	*n* = 27	
Age (year)		53.9 ± 17.2	51.8 ± 13.5	0.627
Gender				
Male	12 (50%)	14 (51.8%)	0.559	
Female	12 (50%)	13 (48.1%)		
BMI		23.0 ± 4.3	25.4 ± 3.6	0.383
Hemodialysis (month)		32.5 ± 25.9	28.1 ± 13.3	0.441
Etiology of ESRD				
Diabetes	12 (50%)	15 (55.5%)	0.645	
Hypertension	6 (25%)	6 (22.2%)		
Polycystic kidney	1 (4.1%)	3 (11.1%)		
Miscellaneous	5 (20.8%)	3 (11.1%)		

**Table 2:** The changes in lipid profile, hemoglobin, and consumed erythropoietin in two groups.

	Carnitine				Placebo			
	*n* = 24				*n* = 27			
	Baseline	8th week	16th week	*P*	Baseline	8th week	16th week	*P*
Triglyceride, mg/dL	165.5 ± 71.3	147.2 ± 60.2	138.3 ± 54.3	0.001	142.2 ± 58.0	161.3 ± 52.4	151.8 ± 48.2	0.029
Cholesterol, mg/dL	147.1 ± 40.8	154.0 ± 30.2	154.5 ± 30.1	0.075	154.8 ± 25.1	155.3 ± 29.3	151.2 ± 24.9	0.517
HD, mg/dL	30.2 ± 6.6	31.0 ± 5.8	34.0 ± 6.6	<0.001	29.5 ± 7.2	30.1 ± 6.1	28.6 ± 8.4	0.185
LDL, mg/dL	84.0 ± 24.7	83.5 ± 20.2	81.0 ± 20.7	0.237	97.6 ± 26.1	92.1 ± 30.0	90.6 ± 28.9	0.227
Hemoglobin, mg/dL	10.5 ± 2.5	10.9 ± 2.4	11.3 ± 2.1	0.081	9.5 ± 2.2	10.1 ± 1.9	9.9 ± 2.5	0.145
Dose of erythropoietin, U/wk	7250 ± 5202	4750 ± 3813	2500 ± 4180	<0.001	8000 ± 3186	6888 ± 4308	6000 ± 5083	0.033

**Table 3:** Comparing lipid profile, hemoglobin, and consumed erythropoietin between two groups.

	Carnitine	Placebo	*P*
	*n* = 24	*n* = 27	
Triglyceride, mg/dL	−31.1 ± 38.7	9.6 ± 32.2	<0.001
Cholesterol, mg/dL	−6.6 ± 16.0	−3.6 ± 24.9	.311
HDL, mg/dL	3.7 ± 2.8	−0.8 ± 3.7	<0.001
LDL, mg/dL	−3.0 ± 10.4	−6.9 ± 24.7	0.239
Hemoglobin, mg/dL	0.7 ± 1.7	0.3 ± 1.4	0.190
Dose of erythropoietin, U/wk	4750 ± 5772	−2000 ± 4296	0.029

**Table 4:** The changes of overall scores of quality of life and its physical and mental subscales in two groups.

	Carnitine				Placebo			
	*n* = 24				*n* = 27			
	Baseline	8th week	16th week	*P*	Baseline	8th week	16th week	*P*
Total SF-36	55.5 ± 24.8	51.7 ± 18.5	55.7 ± 19.4	0.148	38.8 ± 14.0	37.5 ± 14.0	36.2 ± 16.0	0.087
Physical subscales	56.6 ± 23.9	51.2 ± 18.7	52.5 ± 18.0	0.033	36.6 ± 12.1	36.6 ± 12.4	34.5 ± 13.3	0.180
Mental subscales	51.6 ± 24.3	53.8 ± 19.7	58.2 ± 21.2	0.128	42.5 ± 15.1	39.0 ± 14.4	37.6 ± 17.3	<0.001

**Table 5:** Comparison of the changes between scores of quality of life and its physical and mental subscales between two groups.

	Carnitine	Placebo	*P*
	*n* = 24	*n* = 27	
Total SF-36	0.2 ± 12.1	−2.6 ± 7.7	0.152
Physical subscales	−4.1 ± 11.7	8.5 ± 2.1	0.241
Mental subscales	2.1 ± 10.1	−4.6 ± 6.1	0.002
